# Human T Lypmphotrophic Virus (HTLV1) Related Diseases

**Published:** 2011-06-01

**Authors:** S Z Tabei

**Affiliations:** 1 Department of Pathology, Shiraz University of Medical Sciences, Shiraz, Iran

By discovery of HTLV1 virus in 1898 as etiology of the diseases till present, they are considered as the first enemy of human being.[1] Today, advances in immunology have shown that the virus per se may not be the main cause of disease but the interaction between the virus and immune system is important in pathogenesis of the disease. For example in hepatitis B virus after entrance to the hepatocytes, three types of immune reactions may occur including: i). The severe attachment of T lymphocytes[2] to infected hepatocytes that would lead to necrosis and may result into an acute fulfillment of hepatitis, ii). A mild respond of T lymphocytes with minimal injury to hepatocytes as a balance reaction of B lymphocyte would result into a mild hepatitis and nearly a permanent immunity without any chronic disease or hepatocellular carcinoma and iii). Absence or presence of a minimal response that may be prone to hepatocellular carcinomas.

Most treatment modalities are on a basis of suppressing the immune response after administration of steroids or cytotoxic drugs. In the last twenty years, a correlation between psychology and immune system by the axis of hypothalamus was shown. Hypophysis and adrenal glands play an important role in psycho-neuro-endocrino-immunology and treatment of the disease.[3]

The enigma of retrovirus which has a direct correlation with immune system is another interesting point which I would like to discuss briefly here mainly in relation to two articles in the present issue of this journal.

## Biology of the virus

Human T Lypmphotrophic Virus (HTLV) from the family of retroviruses has been divided into three major types of I, II and III.[4] The structure as well as epidemiology of the virus and related diseases have been investigated broadly in the last two decades, both in the world and also in Iran. HTLV1 is a 9 Kb Delta virus that could not be found in the serum of animal as well as human being. It has affinity to T lymphocyte,[5] monocytes, fibroblasts and synovial cells. So the transmission of the virus occurs by an infected cell.[6] Induction of lymphoma and leukemia by HTLV1 seems to happen by a tax protein.[7] The detail mechanism of cancer development could be found in literature.[8]

Epidemiology of the virus after that spread to several areas of the world was considered as endemic regions for HTLV1.[9] Since most studies were undertaken on blood donors of infected persons, the incidence could not be easily determined. In Iran, the prevalence in northeast regions was shown to be 5% which is the highest endemic area in Iran.[10]

## Routes of transmission

The transmission of the virus is vertically from mother to child by blood and milk and can be horizontally by sexual contacts and transfusion of blood or contaminated needle stick too.[11] Sperm and milk can have infected lymphocyte and are important in transmission of the diseases from men to women and mother to child.[12]

## Diseases related to HTLV1

The virus was shown to be a causative agent for several diseases including a) Adult T cell lymphoma leukemia,[13] b) Myelopathic spastic paraparesis,[14] c) Polymysitis[15] and d) Uveitis. The adult T cell lymphoma leukemia mostly occurs in adults and old age and is more common in women than men. The disease is presented by anemia, lymphadenopathy and hypercalcemia. The blast morphology is an especial indicator with a lobular or convoluted nucleus usually called flower cells.[16] Hypercalcemia vastly happens due to TGFB and IL1 which activates osteoclasts and also by PTHr in contaminated cells by HTLV1 too.[17] Tropical paraparesis spastic (TSP) disease is pathologically an infiltration of mononuclear cells into the white matter,[18] degeneration of the Schwann cells and fibrosis of white matter.[19][20]

## Prevention

By considering that the diseases being related to HTLV1 as a chronic and devastating disease, the prevention is important by screening of the blood donors as well as mothers for presence of any antibody against HTLV1 in endemic areas that has an important role in prevention of the disease.

As one of the ways of transmission is by sexual route, mass media education would be important identically done for HIV infection. In Iran, for the first time in 1986, we reported two patients with leukemia from Mashhad in Northeast part of Iran presented with 80% of convoluted blasts in the bone marrow accompanied with hypercalcemia.[21] Although at that time, no virology test was available, by considering blast morphology and hypercalcemia, we thought that the disease may have a correlation with HTLV 1 infection.

In 1992, Sid et al.,[22] also reported four cases who had immigrated from Mashhad to Telaviv and had a positive serology to HTLV1. Two more patients from Mashhad with the diagnosis of multiple sclerosis were shown to be positive for HTLV1 antibody.[23] So, HTLV1 infection was shown to be endemic in Khorasan Province in northeast of Iran. Several surveys by our colleagues in Mashhad University of Medical Sciences confirmed the matter. A survey on blood donors in Khorasan Province and other provinces in the country showed infection in blood samples in Khorasan and other provinces.[24]

In several studies from Iran and Middle East, the high seropositivity with HTLV1 was demonstrated. The retrovirus HTLV I, II and III infections are all tropic to T lymphocytes and microglial cells. Therefore, the immune system in establishment and progress of the diseases and the effect of psychology on the immune system were emphasized.
